# Nomogram model of serum thymidine kinase 1 combined with ultrasonography for prediction of central lymph node metastasis risk in patients with papillary thyroid carcinoma pre-surgery

**DOI:** 10.3389/fendo.2024.1366219

**Published:** 2024-06-03

**Authors:** Xiaolong Song, Sven Skog, Long Wei, Jinlv Qin, Ru Yang, Jin Li, Ji Zhou, Ellen He, Jianping Zhou

**Affiliations:** ^1^ Radioimmunoassay Center, Department of Clinical Laboratory, Shaanxi Provincial People’s Hospital, Xi’an, China; ^2^ Department of Medicine, Shenzhen Ellen-Sven Precision Medicine Institute, Shenzhen, China

**Keywords:** STK1p, ultrasonography, lymph node metastasis, papillary thyroid carcinoma, cancer

## Abstract

**Objective:**

The aim of this study was to develop a nomogram, using serum thymidine kinase 1 protein (STK1p) combined with ultrasonography parameters, to early predict central lymph node metastasis (CLNM) in patients with papillary thyroid carcinoma (PTC) pre-surgery.

**Methods:**

Patients with PTC pre-surgery in January 2021 to February 2023 were divided into three cohorts: the observation cohort (CLNM, *n* = 140), the control cohort (NCLNM, *n* = 128), and the external verification cohort (CLNM, *n* = 50; NCLNM, *n* = 50). STK1p was detected by an enzyme immunodot-blot chemiluminescence analyzer and clinical parameters were evaluated by ultrasonography.

**Results:**

A suitable risk threshold value for STK1p of 1.7 pmol/L was selected for predicting CLNM risk by receiver operating characteristic (ROC) curve analysis. Multivariate analysis identified the following six independent risk factors for CLNM: maximum tumor size >1 cm [odds ratio (OR) = 2.406, 95% confidence interval (CI) (1.279–4.526), *p* = 0.006]; capsule invasion [OR = 2.664, 95% CI (1.324–5.360), *p* = 0.006]; irregular margin [OR = 2.922; 95% CI (1.397–6.111), *p* = 0.004]; CLN flow signal [OR = 3.618, 95% CI (1.631–8.027), *p* = 0.002]; tumor-foci number ≥2 [OR = 4.064, 95% CI (2.102–7.859), *p* < 0.001]; and STK1p ≥1.7 pmol/L [OR = 7.514, 95% CI (3.852–14.660), *p* < 0.001]. The constructed nomogram showed that the area under the ROC curve for the main dataset was 0.867 and that for the validation dataset was 0.830, exhibiting effectivity, and was recalculated to a total score of approximately 383. Through monitoring the response post-surgery, all patients were assessed as tumor-free at 12 months post-surgery, which was significantly associated with a reduction in STK1p to disease-free levels.

**Conclusion:**

We demonstrate for the first time that a novel nomogram including STK1p combined with ultrasonography can assist in the clinical prevention of CLNM, by facilitating timely, individualized prophylactic CLNM dissection, thereby reducing the risk of secondary surgery and the probability of recurrence.

## Highlights

- For the first time, serum thymidine kinase 1 protein (STK1p), a tumor cell proliferating biomarker, was combined with ultrasound parameters to construct a novel nomogram model for the clinical prevention of CLNM in PTC pre-surgery.
- This model will provide a beneficial value for facilitating timely individualized preventive CLNM dissection, thereby reducing the risk of secondary surgery and the probability of recurrence.

## 1 Introduction

Thyroid cancer is a public health problem worldwide, and its incidence is currently significantly increasing (1). Thyroid cell function is closely related to the structure and organization of follicles, and polarization is a key factor in hormone production (2). The most common thyroid cancer is papillary thyroid cancer (PTC), 80%–85% of which derives from well-differentiated follicular thyroid cancer cells and has a good prognosis, and with a 10-year survival of approximately 93%, it is considered an indolent PTC (3); however, differentiated thyroid carcinoma is characterized by an increased number of tumor foci, expanded total tumor diameter, and metastasis or recurrence in lateral lymph nodes (2, 4–7). At initial presentation, PTC is associated with a high rate of lymph node metastases (LNMs), with lateral lymph node metastasis (LLNM) usually occurring after central lymph node metastasis (CLNM). The CLNM is one of the aggressive variants of PTC (3). Preoperative analysis of ultrasonographical features is valuable for predicting PTC with CLNM (4). Furthermore, multifocality and bilaterality can be considered risk factors for CLNM (5), while four or more tumor foci and younger age at LLNM (6), younger age, male sex, tumor size >0.65 cm, and capsular invasion and/or multifocality are associated with high-risk papillary thyroid microcarcinoma (7). Patients with these characteristics are likely to have a poor prognosis and a high mortality rate, and an urgent surgical plan must be arranged (4–7). According to the currently used Tumor–Node–Metastasis staging system (TNM-8) and the previous TNM-7 of PTC, based on a cohort of 1,148 PTC patients, a prognostic value of the disease-free interval (DFI) was estimated. The multivariate analysis showed that advanced tumor stage was an independent risk factor for a lower DFI, but the TNM-7 was most accurate. No molecular parameter can improve the prediction of DFI provided by LNM so far (8).

Conventional ultrasonography (9, 10) and contrast-enhanced ultrasonography (CEUS) (11) are useful tools to characterize whether patients with PTC are at high risk for CLNM, through assessment of CLNM risk factors, including margin, aspect ratio, calcification, loss of echogenic capsule, vascularity, time to enhancement, intensity of enhancement, homogeneity of enhancement, and discontinuous capsule enhancement.

CLNMs are often occult, and pre-surgery examination of patients with PTC by ultrasonography alone may result in a high rate of missed CLNM diagnosis. Whether patients with PTC should undergo prophylactic CLNM dissection and the extent of dissection that is appropriate remain topics of discussion (9, 12, 13).

Nomograms are strongly recommended prognostic/prediction models in medical oncology. These tools allow evaluation of the probability of an individual treatment effect by integrated analyses of different prognostic and determining variables (14). A series of studies have developed nomograms based on ultrasonography characteristics (15, 16) or ultrasonography combined with CEUS for the prediction of CLNM and have shown that they have good predictive value and can assist surgeons in making appropriate surgical decisions for the treatment of individual patients with PTC (17).

Although ultrasonography can be used to observe various biological, morphological, and pathological changes in PTC with CLNM (9–11, 15–17), it cannot assess tumor cell proliferation rate (18). Therefore, it is essential to identify specific molecules that can be used to assess tumor cell proliferation rate, for use in combination with ultrasonography.

Few molecules can serve as serum biomarkers directly related to tumor cell proliferation rate. According to previous basic and clinical studies, thymidine kinase 1 (TK1) is specifically and closely related to the DNA synthesis phase (S-phase) in the eukaryotic cell cycle. TK1 is a unique enzyme that phosphorylates deoxythymine (dThd) to generate deoxythymine monophosphate (dTMP), via the “salvage” pathway, where dTMP serves as a precursor for further phosphorylation during DNA synthesis (18–20). Human TK1 protein concentration and activity in serum are closely related to DNA synthesis (18, 21, 22), and assessment of tumor proliferation rate is recommended in clinical oncology (21–24). A polyclonal antibody (IgY) against a peptide sequence (195–225, GQPAG PDNKE NCPVP GKPGE AVAAR KLFAPQ) toward the C-terminus of human TK1 (TK1-IgY-pAb) has been developed. A convenient enhancing chemiluminescent immune dot blot assay (ECL-dot blot) was used for the detection of serum TK1 protein (STK1p) (21). A series of clinical oncology studies of STK1p, including monitoring of therapeutic effects in patients with 24 types of cancer (bladder, breast, cardiac, cervix, colon, rectum, esophagus, gastric, head & neck, hepatoma, laryngeal, lung, leukemia, lymphoma, myeloma/ multiple myeloma, malignant pleural effusion, nasopharynx, ovarian, mature teratoma ovary, renal cell carcinoma, pancreas, prostate, squamous cell carcinoma, and thyroid) and prognostic assessment in patients with six types of cancer (breast, colon, lung, lymphoma, malignant pleural effusion, and multiple myeloma) (18), as well as analysis of risk processes during early tumorigenesis in 160,068 routine health screens, have been conducted (24). It demonstrated that the STK1p is a reliable tumor proliferating biomarker for the assessment of pre/early cancerous progression in routine health screening, as well as monitoring effect and prognosis in clinical oncology. However, a nomogram model performing individualized risk probability progression is still lacking.

An aggressive variant (T1–2N1) carries a worse prognosis than the indolent tumor (T1–2N0) of PTC. It is important to study protein molecules/genes associated with tumor proliferation as well as morphological characteristics of these two types, which will provide vital assistance in establishing early risk prediction model, evaluating the effectiveness of early effective treatment interventions in patients, and improving individualized survival

Here, for the first time, we constructed a nomogram model combining STK1p and five ultrasonography parameters as independent factors for the early prediction of the risk of CLNM in patients with PTC. This novel model can be used to guide clinicians in performing individualized prophylactic CLNM dissection, to reduce the risk of secondary surgery and probability of recurrence.

## 2 Patients and methods

### 2.1 Research design

To design a nomogram model combining STK1p and ultrasound characteristics to predict the risk of CLNM (T1N1M0–T2N1M0) in patients with PTC, (1) a receiver operating characteristic (ROC) curve analysis was first conducted, to select a suitable risk threshold of STK1p for the prediction of CLNM; (2) multivariate logistic regression was applied, to determine whether STK1p and ultrasound parameters can serve as clinical risk factors for the prediction of CLNM; (3) a nomogram predicting the probability of CLNM was constructed using R 3.5.3. (www.r-project.org); (4) the response to surgery was monitored to determine the detection of STK1p pre- and post-surgery (3–12 months) in patients with and without CLNM (NCLNM, T1N0M0–T2N0M0); and (5) according to the evaluation criteria for the efficacy of the treatment of the solid tumors, the postoperative patients were evaluated every 6 months.

### 2.2 Patients

This was a prospective study. Preliminary evaluation of a cohort of patients with PTC (*n* = 476) was conducted pre-surgery by conventional ultrasonography at Shaanxi Provincial People’s Hospital, from January 2021 to February 2023.

The function of human TK1 is for the reliable assessment of cell proliferating rate, including both normal and tumor proliferating cells (18–21). How do we know that STK1 reflects the proliferation rate of the tumor cells, making it possible to assess the treatment effect or prognosis of PTC? To realize this, it is necessary to exclude a range of factors associated with cell proliferation.

The inclusion and exclusion criteria are as follows:

Inclusion criteria.

According to the diagnostic criteria for PTC (25), 368 PTC tumors were diagnosed by pathology and patients were then clinically selected for inclusion into three cohorts: (1) the observation cohort (aggressive behavior, CLNM, *n* = 140, T1N1M0–T2N1M0); (2) the control cohort (indolent behavior, NCLNM, *n* = 128, T1N0M0–T2N0M0); and (3) the external validation cohort, with cases (CLNM, T1N1M0–T2N1M0, *n* = 50) and controls (NCLNM, T1N0M0–T2N0M0, *n* = 50).

Exclusion criteria.

The exclusion criteria followed the specific normative STK1 data for health screening (26): (1) medical history of any other benign/pre-malignant/malignant tumors; (2) any type of virus infection; (3) long-term chronic inflammation, or acute inflammation within 4 weeks; (4) obese or extremely thin subjects with a body mass index (BMI) above 28.0 kg/m^2^ or below 18.5 kg/m^2^; and (5) elevated STK1p by any medication (e.g., exogenous hormone therapy), temporary physiological emergency response caused by excessive fatigue, or women who were pregnant or on their menstrual cycle. It was found that the transiently elevated STK1 values in patients of post-operative treatment might be due to surgery-induced complications, such as anemia and infection/inflammation, as well as to operation execution times and age of patients. The detection of STK1p should not be used within 1–3 weeks to avoid non-tumor-related increases in STK1, and thus misleading results (27).

**Figure 1:**
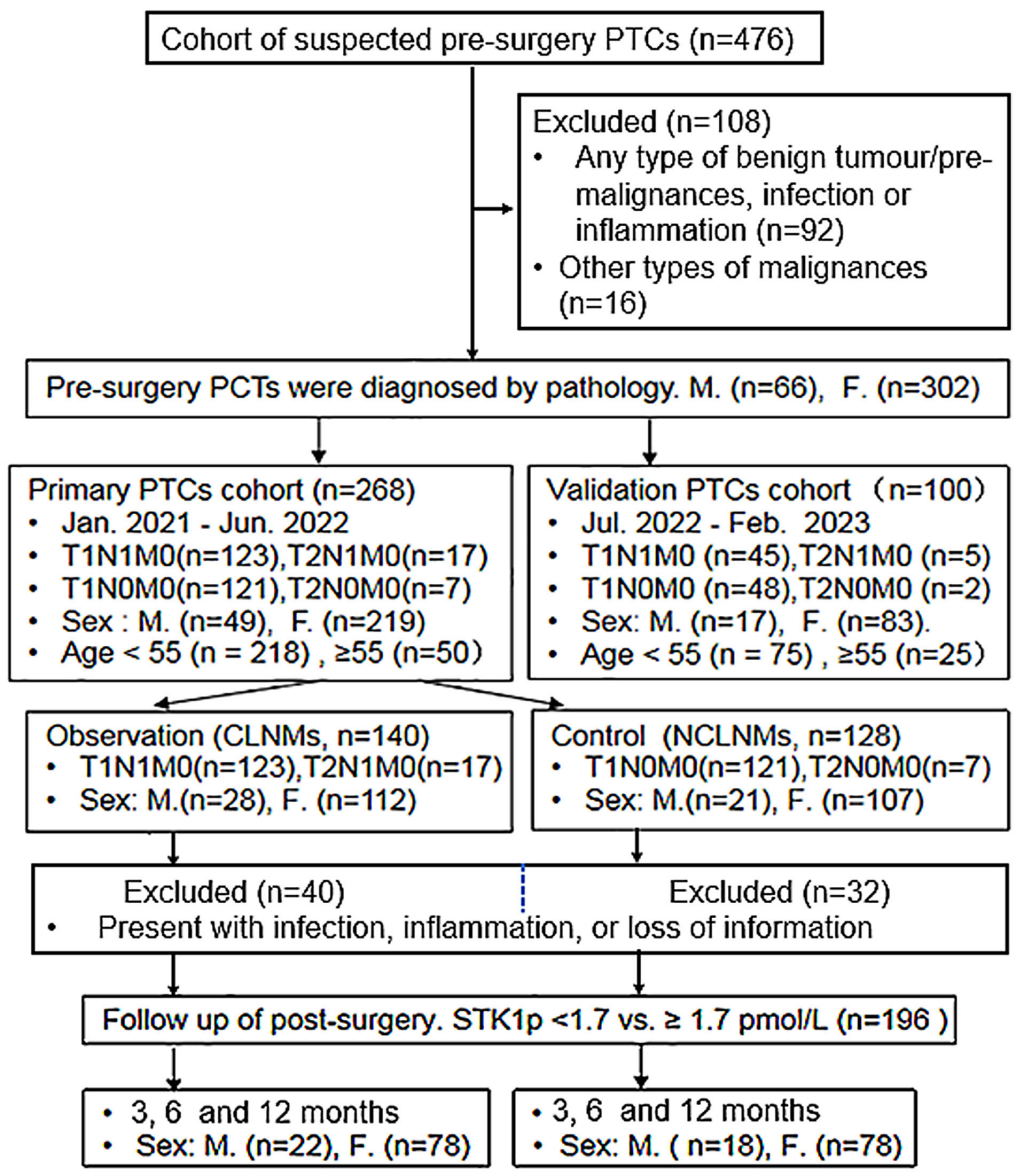
A brief flowchart of the selection process for these cohorts.

A brief flowchart of the selection process for these cohorts is presented in **Figure 1**.

### 2.3 Analysis of clinical ultrasonography data

Evaluation of ultrasonography parameters was carried out independently by two experienced physicians, according to the 2018 TI-RADS Guidelines (28).

### 2.4 Patient treatment and follow-up

Prophylactic PTC dissection was performed for all patients who underwent partial or total thyroidectomy, and central lymph node dissection, while preserving the recurrent laryngeal nerve and parathyroid gland. Patients with CLNM underwent thyroid-stimulating hormone (TSH) suppression and iodine therapies.

Follow-up: (1) Post-surgery PTC was confirmed by ultrasound and pathology, divided into partial or total thyroidectomy and central lymph node dissection; (2) thyrotropin suppression therapy and/or ^131^I therapy post-surgery followed the standard treatment plan, and the postoperative patients were retested regularly (once every 3 months); (3) STK1p was determined pre-/post-surgery for 3, 6, and 12 months; and (4) according to the Response Evaluation Criteria in Solid Tumors (29), each patient was scored as having complete response (CR), partial response, stable disease, or progressive disease. STK1p assay was used to monitor therapeutic effect at 6 and 12 months post-surgery in the observation and control cohorts.

### 2.5 Serological assay

Fasting venous blood samples (3 mL) were collected from patients in the morning before surgery, and the serum was separated by centrifugation at 3,000 rpm for 5 min and analyzed within 3 h or stored at –20°C. Data analysis was completed within 1 week. Each serum specimen should be tested at least twice with an SD error of less than 10%.

#### 2.5.1 STK1p assay

Serum specimens were probed with human TK1-IgY-pAb by STK1p analysis kit for the detection of serum TK1 protein (STK1p) based on a convenient enhancing chemiluminescent immune dot blot assay (ECL-dot blot, Sino-Swed Tongkang Bio-Tech. Ltd., Shenzhen, China; www.sstkbiotech.com). Briefly, 3 μL of serum specimens was directly applied to nitrocellulose membranes (NCs) in duplicate. The TK1 calibrators were also dotted onto the membranes at different concentrations (2.2, 6.6, and 20 pmol/L) as an extrapolated standard. According to the manufacturer’s instruction, the following protocol is performed in an incubator: (1) place the NC with the dotted samples in a reaction tray and add the block buffer for 30 min, with slight agitation; (2) add the anti-TK1-IgY with 1:500 dilution buffer and incubate for 2 h at room temperature, with slight agitation; (3) wash with wash buffer 3 × 5 min, with slight agitation; (4) add biotinylated secondary antibody with 1:500 dilution buffer for 1 h at room temperature, with slight agitation; (5) continue washing, see step 3; (6) add streptavidin-horseradish peroxidase with 1:1,500 dilution buffer and incubate for 1 h at room temperature, with slight agitation; (7) continue washing, see step 3. Finally, place the NC into the provided plastic wrap in the kit and add ECL substrates of A and B for 1 min accurately, and then remove the excess reagent with a moisture absorbent paper and continue the reaction for 5 min. The intensity of spots on the NC was determined using a CIS-l Imaging System (Sino-Swed Tongkang Bio-Tech Inc., Shenzhen, China). The STK1p value of each spot was calculated and expressed as pmol/L, based on the TK1 calibrators.

#### 2.5.2 Detection of TSH and TGAb

TSH (thyrotropin) and TGAb (thyroglobulin antibody) were detected using an automatic chemiluminescence immunoanalyzer and corresponding kits [Autobio Diagnostics Co., Ltd. (AutoLumo A2000), Zhengzhou, China].

The TSH assay is based on the one-step sandwich of the combined TSH monoclonal antibodies. Briefly, (1) aspirate and transfer 100 µl of serum sample and 100 µl of TSH calibrators into the reaction vessel, respectively; (2) add 20 μl of the coated TSH monoclonal antibody 1 to the microparticles, and then add 50 μl of assay buffer and 50 μl of enzyme-linked TSH antibody 2; (3) incubate at 37°C for 34 min for immunological reaction; (4) wash the reaction mixture by cleaning buffer 5 times for 18 s; and (5) add chemiluminescent substrates of A (50 μl) and B (50 μl) and mix well. The serum TSH was detected by luminescence intensity in 90 s and was expressed as µIU/mL, based on the TSH calibration.

The TGAb assay is based on the two-step indirect method. Briefly, (1) aspirate and transfer 10 μl of serum sample with 90 μl of dilution buffer and 100 μl of TGAb calibrators into the reaction vessel; (2) add 20 μl of the microparticle solution to the reaction vessel; (3) mix the reaction solution and incubated at 37°C for 15 min; (4) wash the reaction mixture with cleaning buffer five times for 18 s; (5) add 100 μl of enzyme-linked anti-IgG human antibody, mix well, and incubate at 37°C for 17 min; (6) wash the reaction mixture with cleaning buffer five times for 18 s; (7) add chemiluminescent substrates of A (50 μl) and B (50 μl), respectively, and mix well. The serum TGAb was detected by luminescence intensity in 90 s and was expressed as IU/mL based on the TGAb calibration.

### 2.6 Statistical analysis

IBM SPSS Statistics (version 25.0, USA) was used for all data analyses, including logistic regression, *t*-test, *Z*-test, Pearson chi-square test, ANOVA, and ROC curves. *p* < 0.05 was considered significant. Nomograms were evaluated for discrimination ability using the consistency index and area under the ROC curve (AUC) values. Depending on the aim of the study, different statistical limitations were used (see the Results part). Nomogram construction and subsequent validation were performed in R 3.5.3 (www.r-project.org). AUC >0.75 was considered significant (14).

## 3 Results

### 3.1 Can the combination of STK1p and ultrasonography parameters improve risk assessment for CLNM in PTC patients?

**Figure 2:**
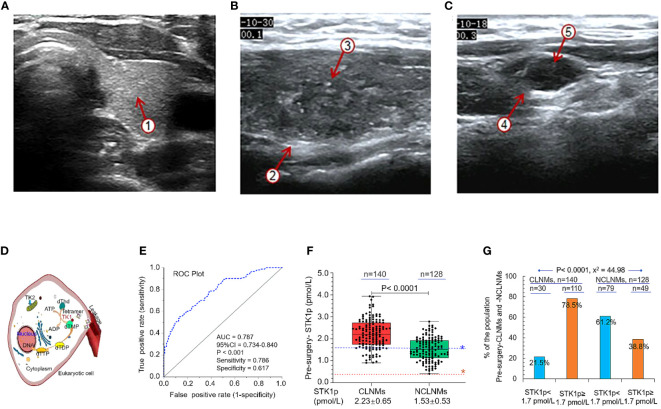
An example of ultrasonography. **(A)** Normal thyroid tissue with a clear boundary, regular and complete capsule, medium echo, and evenly distributed fine and dense light spots ①. **(B)** Non-central lymphoid metastasis tissue with unclear boundaries, and an irregular margin ②, with multiple strong echoic small calcifications ③. **(C)** Central lymph node metastasis tissue with round, hypoechoic nodules, an unclear boundary between the cortex ④ and medulla, disappearance of the lymphatic portal, slightly full shape, and multiple strong echoic small calcifications ⑤. **(D)** Schematic diagram showing the function of TK1 in eukaryotic cell DNA synthesis. **(E)** ROC curve analysis to determine the STK1p threshold value in patients with papillary thyroid carcinoma. **(F)** STK1p levels in patients with (CLNM) and without (NCLNM) central lymph node metastasis. **(G)** Percentages of patients with STK1p levels < and ≥ 1.7 pmol/L. Threshold value of STK1p = 0.38 pmol/L for disease-free healthy individuals (19). Risk threshold value of STK1p = 1.7 pmol/L was selected for patients with CLNM. Pearson chi-square test, *p* < 0.001, *χ* ^2^ = 44.98.

The results of applying a combination of STK1p and ultrasonography parameters to assess the risk process for CLNM in patients with PTC are presented in **Table 1**; **Figures 2A–G**.

**Table 1:** Comparative analysis of individual factors in patients with papillary thyroid carcinoma with and without central lymph node metastasis[n (%)].

Characteristics	CLNM(n=140)	NCLNM(n=128)	x^2^/t/Z	*P*	OR	OR(95%CI)
Age (year)						
≥ 55	23(16.4)	27(21.1)	0.959	0.327	0.735	0.397-1.362
< 55	117(83.6)	101(78.9)				
Gender			0.578	0.447	0.785	0.420-1.466
Male	28(20.0)	21(16.4)				
Female	112(80.0)	107(83.6)				
Nodular goiter			0.165	0.68	0.901	0.545-1.490
Presence	47(33.6)	46(35.9)				
Absence	93(66.4)	82(64.1)				
Echogenic foci			5.496	0.019	1.803	1.099-2.958
Presence	94(67.1)	68(53.1)				
Absence	46(32.9)	60(46.9)				
Echogenicity			0.368	0.544	0.849	0.500-1.441
Hypoechoic	97(69.3)	93(72.7)				
Hyper- or equal-echogenicity	43(30.7)	35(27.3)				
Margin			11.486	0.001	2.665	1.496-4.745
Irregular	117(86.3)	84(65.6)				
Regular	23(16.4)	44(34.4)				
Aspect ratio			2.005	0.157	1.505	0.853-2.656
> 1	112(80.0)	93(72.7)				
≤ 1	28(20.0)	35(27.3)				
Tumor foci number			17.325	<0.001	2.871	1.736-4.747
≥ 2	78(55.7)	39(30.5)				
< 2	62(44.3)	89(69.5)				
Position			4.602	0.203	–	–
Upper pole	28(20.0)	36(28.1)				
Middle part	26(18.6)	29(22.7)				
Lower pole	59(42.1)	40(31.3)				
Isthmus	27(19.3)	23(18.0)				
Maximum tumor size (cm)			18.130	<0.001	2.933	1.77
> 1	80(57.1)	40(31.3)				
≤ 1	60(42.9)	88(68.8)				
Capsule invasion			34.173	<0.001	4.625	2.728-7.841
Presence	108(77.1)	54(42.2)				
Obscene	32(22.9)	74(57.8)				
Maximum CLN size (cm)			2.062	0.151	1.422	0.879-2.302
> 1	79(56.4)	61(47.7)				
≤ 1	61(43.6)	67(52.3)				
CLN flow signal			30.347	<0.001	5.000	2.746-9.105
Presence	63(45.0)	18(14.1)				
Obscene	77(55.0)	110(85.9)				
TSH (uIU/mL)	2.85±1.71	2.93±1.66	0.395	0.693	–	–
TgAb (IU/mL)	10.00 (10.00,70.07)	10.00 (10.00,96.39)	1.097	0.273	–	–
STK1p (pmol/L)	2.22±0.65	1.53±0.53	9.495	<0.001	–	–

Ultrasonography is useful for observing the series of characteristic changes that occur during progression from thyroid tissue in a normal thyroid to NCLNM and CLNM; examples are illustrated in **Figures 2A–C**, respectively.

Nevertheless, it is challenging to assess the evolution of individual differences in proliferation rate between CLNM (observations: aggressive behavior, T1N1M0–T2N1M0, *n* = 140) and NCLNM (controls: indolent behavior, T1N0M0–T2N0M0, *n* = 128).

In this study, we attempted to use the STK1p, a tumor proliferating serum biomarker, combined with ultrasonography, to construct a nomogram model.

As mentioned in the Introduction, TK1 is closely related to the rate of DNA synthesis (S-phase) in the cell cycle in eukaryotic cells. **Figure 2D** is a schematic diagram showing the mechanism by which the tetrameric form of TK1 catalyzes the conversion of dThd to dTMP, and further conversion to thymidine triphosphate, finally leading to DNA synthesis in eukaryotic cells (18–20). TK1 released from cells can be measured in serum samples, providing convenient, non-invasive detection of the active functional tetrameric form of TK1, which corresponds to the physiological cell proliferation rate (30).

Comparing to individual factors between CLNM and NCLNM groups, it revealed a statistically significant high value of six factors in CLNMs (**Table 1**, all *p* < 0.05): echogenic foci presence vs. absence, margin (irregular vs. regular), tumor number (≥2 vs. <2), maximum tumor size (cm, >1 vs. ≤1), capsule invasion (presence vs. absence), and CLN flow signal (presence vs. absence). It is particularly noted that the STK1p pre-surgery value showed a statistically significant difference (*p* < 0.001) between the CLNM (STK1p: mean value 2.22 ± 0.65 pmol/L) and the NCLNM (STK1p: mean value 1.53 ± 0.53 pmol/L) groups, indicating that the significant increase of the STK1p value was closely related to rapid proliferating tumor cells in the CLNM group, while the relatively low STK1p value was closely related to slow proliferating tumor cells in the NCLNM group; even the CLNM and NCLNM belonged to the same PTC group. It supports the idea that CLNM is a more aggressive type of PTC compared to the NCLNM type (3). Therefore, it is possible to use ROC curve analysis of the STK1p data from the CLNM (observations) and the NCLNM (controls) to determine the risk threshold of STK1p for predicting CLNM.

The AUC value for the ROC curve analysis of STK1p pre-surgery was 0.787 (*p* < 0.001, **Figure 2E**). According to the maximum Jorden index value (31), sensitivity and specificity were 78.6% and 61.7%, respectively. An appropriate predicted CLNM risk threshold value for STK1p of 1.7 pmol/L was selected. The STK1p level between the two groups of CLNM and NCLNM was further investigated by comparative analysis. Interestingly, we found that STK1p levels in patients with CLNM were significantly higher than those in the NCLNM control group (**Figure 2F**; *p* < 0.001). Next, patients in the CLNM and NCLNM groups were significantly stratified according to STK1p level (<1.7 and ≥1.7 pmol/L). Approximately 75% of patients in the CLNM group had high STK1p (≥1.7 pmol/L), whereas only 38.6% of patients in the NCLNM group had high STK1p (**Figure 2G**; Pearson chi-square test, *p* < 0.001, *χ* ^2^ = 44.98).

Since both the observation (CLNM) and control (NCLNM) cohorts belonged to patients with PTC, we considered that the AUC value of 0.787 (*p* < 0.001) should be expected correctly with statistical significance (0.7 > AUC <0.8) (32). With the maximum Jorden index (31), the sensitivity and specificity were 78.6% and 61.7%, respectively; thus, we can set an optimal STK1p cutoff value to assess a risk process for CLNM patients.

However, age, TSH, TGAb, sex, nodular goiter, echogenicity, aspect ratio, and position did not differ significantly between the CLNM and NCLNM groups (all *p* > 0.05, **Table 1**).

The preliminary summary of the combination of the STK1p value and the six ultrasound parameters improved the risk assessment for patients with CLNM.

### 3.2 Construction of a nomogram risk prediction model for CLNM

#### 3.2.1 Multivariate logistic regression analysis

The results of multivariate analysis are presented in **Table 2**. Six risk factors were significantly and independently associated with CLNM patients: maximum tumor size ≥1 cm (*p* = 0.06); capsule invasion (*p* = 0.06); irregular margin (*p* = 0.004); CLN flow signal (*p* = 0.002); tumor-foci number ≥2 (*p* < 0.001); and STK1p ≥1.7 pmol/L (*p* < 0.001).

**Table 2:** Multivariate logistic regression analysis of factors predicting central lymph node metastasis risk in patients with papillary thyroid carcinoma.

Characteristics	B	S.E.	Wald	Sig.	OR	OR (95%CI)
STK1p ≥1.7 pmol/L	2.017	0.341	34.989	<0.001	7.51	3.852-14.660
Irregular margin	1.072	0.376	8.112	0.004	2.92	1.397-6.111
Tumor foci numbers ≥2	1.402	0.336	17.368	<0.001	4.06	2.102-7.859
Maximum tumor size >1cm	0.878	0.322	7.421	0.006	2.41	1.279-4.526
Capsule invasion	0.98	0.357	7.538	0.006	2.66	1.324-5.360
CLN flow signal	1.286	0.407	10.004	0.002	3.62	1.631-8.027
Constant	-3.825	0.525	53.147	<0.001	–	–

OR, odds ratio; CI, confidence interval.

#### 3.2.2 Verification of the clinical characteristics of cases and controls

Five clinical characteristics significantly predicted risk of aggressive CLNM in the validation cohort, namely, echogenic foci (presence vs. absence, *p* = 0.035), hypoechoic echogenicity (hyper- vs. equal echogenicity, *p* = 0.041), margin (irregular vs. regular, *p* = 0.006), CLN flow signal (presence vs. absence, *p* = 0.002), and STK1p (between CLNM and NCLNM, *p* < 0.001) (**Table 3**, *p* < 0.05).

**Table 3:** Clinical characteristics of the patients with and without central lymph node metastasis in the validation cohort(n (%)).

Characteristics	CLNM(n=50)	NCLNM(n=50)	x^2^/t/Z	*P*	OR	OR(95%CI)
Age (year)						
≥ 55	10(20.0)	15(30.0)	1.333	0.248	0.583	0.233-1.463
< 55	40(80.0)	35(70.0)				
Gender			0.638	0.424	0.651	0.226-1.875
Male	10(20.0)	7(14.0)				
Female	40(80.0)	43(86.0)				
Nodular goiter			0.762	0.383	0.682	0.288-1.614
Presence	13(26.0)	17(34.0)				
Absence	37(74.0)	33(66.0)				
Echogenic foci			4.456	0.035	2.488	1.057-5.857
Presence	38(76.0)	28(56.0)				
Absence	12(24.0)	22(44.0)				
Echogenicity			4.167	0.041	2.333	1.027-5.300
Hypoechoic	35(70.0)	25(50.0)				
Hyper- or equal-echogenicity	15(30.0)	25(50.0)				
Margin			7.429	0.006	3.273	1.372-7.806
Irregular	39(78.0)	26(52.0)				
Regular	11(22.0)	24(48.0)				
Aspect ratio			1.099	0.295	1.556	0.679-3.561
> 1	35(70.0)	30(60.0)				
≤ 1	15(30.0)	20(40.0)				
Tumor foci number			1.004	0.316	1.496	0.679-3.294
≥ 2	26(52.0)	21(42.0)				
< 2	24(48.0)	29(58.0)				
Position			7.027	0.071	–	–
Upper pole	7(14.0)	15(30.0)				
Middle part	8(16.0)	12(24.0)				
Lower pole	24(48.0)	13(26.0)				
Isthmus	11(22.0)	10(20.0)				
Maximum tumor size (cm)			0.762	0.383	0.682	0.288-1.614
> 1	13(26.0)	17(34.0)				
≤ 1	37(74.0)	33(66.0)				
Capsule invasion			1.999	0.157	1.778	0.798-3.958
Presence	25(50.0)	18(36.0)				
Obscene	25(50.0)	32(64.0)				
Maximum CLN size (cm)			0.644	0.422	0.724	0.329-1.594
> 1	21(42.0)	25(50.0)				
≤ 1	29(58.0)	25(50.0)				
CLN flow signal			9.333	0.002	4.125	1.611-10.559
Presence	22(44.0)	8(16.0)				
Obscene	28(56.0)	42(84.0)				
TSH (uIU/mL)	3.04±1.85	2.76±1.73	0.810	0.420	–	–
TgAb (IU/mL)	10.00 (10.00,65.00)	10.0 (10.00,72.79)	0.234	0.815	–	–
STK1p (pmol/L)	1.93±0.68	1.54±0.58	3.110	0.002	–	–

#### 3.2.3 Performance of the nomogram for predicting CLNM

**Figure 3:**
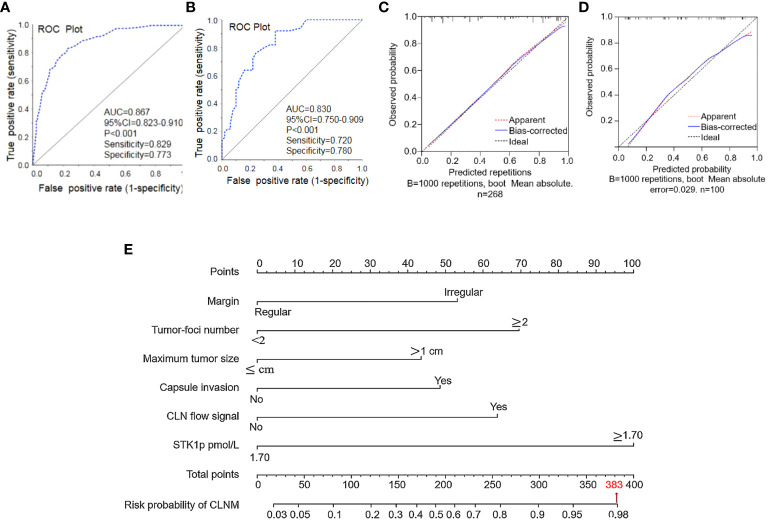
Performance of the nomogram for predicting central lymph node metastasis (CLNM). **(A)** Nomogram prediction model and **(B)** validation of the model by ROC curve analysis. Prediction calibration curve analysis of the training **(C)** and validation **(D)** cohorts, according to calibration curves based on the nomogram model in the training and validation cohorts. Nomogram for predicting the probability of CLNM in patients with papillary thyroid carcinoma **(E)**. Performance of the nomogram. AUC, area under the curve; ROC, receiver operating characteristic curve.

The results of assessment of the performance of our nomogram for predicting CLNM in patients with PTC are presented in **Figures 3A–E**. Based on the results of multivariate analysis (**Table 2**) and patient clinical characteristics (**Table 3**), the AUC of the ROC curve for the main dataset was 0.867 (**Figure 3A**) and that for the validation dataset was 0.819 (**Figure 3B**), indicating good agreement and high significance (14). The calibration curves for the training cohort showed an average absolute error of 0.010 (**Figure 3C**), while that for the verification cohort showed an average absolute error of 0.029 (**Figure 3D**), confirming good agreement between the predicted value and the actual pathological results. Finally, by STK1p combined with ultrasonography (margin, tumor foci number, maximum tumor size, capsule invasion, and CLN flow signal), we constructed a nomogram model to predict CLNM risk and recalculated a total score of about 383 (**Figure 3E**).

#### 3.2.4 STK1p monitoring pre- and post-surgery (3–12 months) in patients with and without CLNM

**Figure 4:**
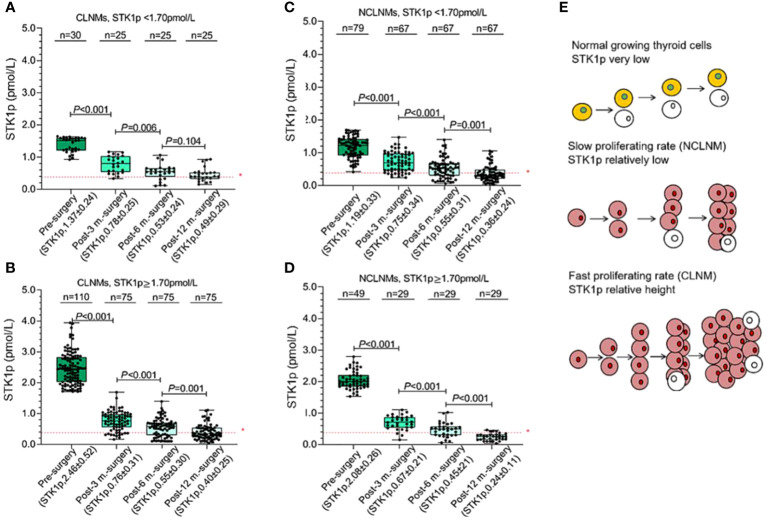
Monitoring of STK1p levels pre-surgery and at 3, 6, and 12 months post-surgery. **(A, B)** STK1p < 1.70 pmol/L vs. STK1p ≥ 1.70 pmol/L in the central lymph node metastasis (CLNM group). **(C, D)** STK1p < 1.70 pmol/L vs. STK1p ≥ 1.70 pmol/L in the control (NCLNM) group. **(E)** Schematic diagram of thyroid cell proliferation rates in relation to STK1p level. *Threshold value of STK1p = 0.38 pmol/L for a disease-free individual (19).

In this study, all patients were evaluated 6–12 months post-surgery as having a CR (i.e., they were tumor-free). We measured STK1p levels of patients pre-surgery and at 3–12 months post-surgery in both the CLNM and NCLNM groups (**Figure 4**). Pre-surgery STK1p levels were significantly higher than those post-surgery (*p* < 0.01), which returned to expected levels for healthy disease-free (mean value of STK1p = 0.38 pmol/L) (18) or tumor-free/proliferating disease-free (mean value of STK1p = 0.38 pmol/L) (26) at 3, 6, and 12 months post-surgery, particularly in patients with STK1p levels ≥1.7 pmol/L pre-surgery; the lowest detected value of STK1p was 0.02 mol/L (**Figures 4A–D**).

A schematic diagram is presented in **Figure 4E**, showing the slow and fast tumor proliferation rates and differences in STK1p levels in the NCLNM and CLNM groups, respectively.

## 4 Discussion

Although a series of reports have shown that nomograms based on risk factors determined from ultrasonography or CEUS parameters can be used to evaluate the probability of individual treatment effect in PTC patients with CLNM (4–7, 15–17, 33, 34), those characteristics do not measure PTC cell proliferation rate. In this study, we constructed a nomogram prediction model for CLNM, based on ROC curve and multivariate logistic regression analyses. The AUC for the ROC curve was 0.787 in the primary model, and a suitable risk threshold for STK1p level of 1.7 pmol/L was selected for predicting risk in patients with aggressive CLNM. Multivariate logistic regression analysis identified six significant independent clinical risk factors, namely, maximum tumor size ≥1 cm [odds ratio (OR) = 2.406; 95% confidence interval (CI) (2.102–7.859), *p* = 0.006]; capsule invasion [OR = 2.664, 95% CI (1.324–5.360), *p* = 0.006]; irregular margin [OR = 2.922; 95% CI (1.397–6.111), *p* = 0.004]; CLN flow signal [OR = 3.618, 95% CI (1.631–8.027), *p* = 0.002]; tumor-foci number ≥2 [OR = 4.064, 95% CI (2.102–7.859), *p* < 0.001]; and STK1p ≥1.7 pmol/L [OR = 7.514, 95% CI (3.852–14.660), *p* < 0.001]. These risk factors were incorporated into the nomogram, which had AUC values for the main dataset and validation dataset of 0.867 and 0.830, respectively. The nomogram for predicting the probability of CLNM was constructed. A total score of approximately 383 was recalculated. The six factors associated with high risk of CLNM were ordered as follows: maximum tumor size < capsule invasion < irregular margin < CLN flow signal < tumor-foci number < STK1p ≥1.7 pmol/L.

In this study, we found that unclear boundaries, nodular microcalcification, tumor-foci numbers, tumor diameter, and lymph node blood-flow signal were independent risk factors for CLNM, consistent with the findings of previous studies (4–7, 15–17, 33, 34). All patients were assessed as tumor-free at 12 months post-surgery, which was significantly associated with a reduction in STK1p to disease-free levels (**Figures 4A–D**), indicating that prophylactic CLNM dissection, based on individual patient monitoring, was effective. Thus, our novel nomogram, incorporating STK1p and ultrasonography characteristics, is a favorable predictive model to inform timely decisions on individualized prophylactic CLNM dissection, reducing the risk of secondary surgery and the probability of recurrence. We consider that STK1p combined with ultrasonography is a potentially important factor for evaluation of CLNM aggressive behavior, which can facilitate individualized clinical monitoring and prognosis prediction.

Human TK1 is a key S-phase specific enzyme involved in DNA synthesis during the cell cycle. The tetrameric form of TK1 is released from cells into the serum, where it rapidly binds to a variety of serum proteins via S-S bridges, to form a natural macromolecule protein complex (approximately 730 kDa) that exhibits high stability in the serum (30). The non-invasive STK1 assay of tumor proliferation rate has attracted considerable attention. Not all TK1 serum biomarkers are suitable for assessment of early tumor proliferation rates in a clinical oncology setting (18). First, different TK1 biomarkers rely on appropriate antibody preparation. The selection of key peptides (epitopes) related to the proliferation function of TK1 in the human cell cycle is important, and 31 peptides near the C-terminus of human TK1 have been identified as an excellent immune antigen. Second, the selection of animal for antibody preparation is also very important. Based on genetic differences, IgY-TK1 antibody prepared from chicken can effectively reduce non-specific immune cross-reaction, compared with IgG-TK1 antibody prepared from mouse. The sensitivity of the detection method is crucial. The ECL-dot blot assay is highly sensitive, detecting as little as 0.01 pmol/L STK1p, which is approximately 120 times more sensitive (18, 35) than a TK1 enzyme-linked immunosorbent assay (36), which can detect invisible malignant tumors (35). In this study, we demonstrated that the methodology of STK1p using TK1-IgY-pAb on the ECL-dot blot platform (21) can serve as assessment of the CLNM proliferating rate. As shown in **Figures 4C,D**, the detected minimum STK1p level was 0.02 pmol/L, which is consistent with the verified lowest detectable value of STK1p (0.01 pmol/L) (18, 35) in accordance to the limit of blank, limit of detection, and limit of quantitation (37).

The regulation of normal cell growth and unlimited tumor cell proliferation involves not only control of cell growth, but also apoptosis regulation (19, 38). In disease-free individuals, STK1p levels are relatively low (18). In contrast, once multiple genetic mutations occur, resulting in enzyme deficiencies and metabolic disorders, including resistance to apoptosis and the ability to evade the immune system, unlimited growth occurs (18, 38). Increased STK1p levels are reported to be correlated with clinical tumor stage I–III in patients with thyroid cancer (39). In this study, we demonstrate for the first time that STK1p levels are significantly higher in patients with CLNM than in those without (NCLNM; *p* < 0.0001) (**Figure 4F**). Furthermore, the percentage of patients in the CLNM group with high STK1p (≥ 1.7 pmol/L) was 78.5%, compared with only 38.6% in the NCLNM group (**Figure 2G**). Based on these findings, we consider STK1p to be a reliable biomarker for distinguishing relatively slow proliferation rates in patients without CLNM from the more rapid proliferation rate in those with CLNM (**Figure 4E**). Analysis of STK1p in the CLNM and NCLNM groups at 6 and 12 months post-surgery demonstrated that levels were significantly reduced and comparable to those in healthy individuals (mean value of STK1p = 0.38 pmol/L, **Figures 4C, D**) (18, 26), indicating successful prophylactic dissection in all patients with PTC. Furthermore, it was observed that the STK1p returned to the normal level after post-surgery patients with tumour free, indicating that metabolic regulation in cells had entered in the normal apoptotic pathway. Our study supports the findings of previous basic studies from 1960 (40) to the present day (18).

TSH and TGAb can be used to assess the effect of treatment for CLNM. TSH is a glycoprotein hormone secreted by the pituitary gland, and TSH-receptor is a G-protein-coupled receptor located on the membrane of thyroid follicular epithelial cells. When combined with the TSH-receptor, TSH can stimulate thyroid tumor growth (41) and increase the incidence of CLNM (42). TGAb is a thyroid autoantibody and an important index for diagnosis of autoimmune thyroiditis (43). Elevated serum TGAb levels are related to autoimmune thyroiditis and thyroid cancer (44). Our results showed that levels of TSH and TGAb were not correlated with CLNM and NCLNM in patients with PTC pre-surgery, suggesting that validation of basic studies is necessary, and that serum TSH, TG, and TGAb levels are unlikely to be useful for direct assessment of the cell proliferation rate in patients with and without CLNM.

Different findings regarding the relationship between age and CLNM have been reported; for example, one study reported that age ≥55 years was a risk factor for CLNM (45), whereas another found no significant association with age or sex (46). Age ≥55 years was not significantly associated with CLNM in our study. These differences may be related to the fact that most studies are retrospective analyses, leading to selection bias in study subjects.

In this study, for the first time, we combined STK1p levels with ultrasound parameters to construct a nomogram for the clinical prevention of CLNM. All patients were evaluated as tumor-free at 12 months post-surgery. With individualized monitoring to assess therapeutic effects, we found that serum STK1p in tumor-free individuals decreased significantly to levels seen in healthy, disease-free people. Nevertheless, this was a single-center study, and the data included in the training and validation cohorts were limited. In the future, large-sample, multi-center studies, with long-term follow-up to assess prognosis should be conducted.

## 5 Conclusion

We describe the development of a novel nomogram, combining STK1p levels with ultrasonography parameters, to predict the risk of CLNM in patients with PTC. Factors included in the nomogram were arranged as follows: maximum tumor size < capsule invasion < irregular margin < CLN flow signal < tumor-foci number < STK1p ≥1.7 pmol/L. The model is suitable for predicting risk processes for aggressive CLNM. With individualized monitoring to assess therapeutic effects, all patients were evaluated as tumor-free at 12 months post-surgery, and the STK1p was significantly decreased to levels associated with healthy, disease-free status. This novel nomogram will be of benefit in guiding individualized clinical decisions on the timely performance of prophylactic CLNM dissection, to reduce the risk of secondary surgery and the probability of local recurrence.

## Data availability statement

The original contributions presented in the study are included in the article/supplementary material. Further inquiries can be directed to the corresponding authors.

## Ethics statement

This clinical study was approved by the ethics committee of the Shaanxi Provincial People’s Hospital, Xi’an, China (No. 2020B001). The studies were conducted in accordance with the local legislation and institutional requirements. The patient signed the written informed consent, but the next of kin or legal guardian is not required to sign because this clinical study of Serum Thymidine Kinase 1 Combined with Ultrasonography for Prediction of Central Lymph Node Metastasis Risk in Patients with Papillary Thyroid Carcinoma Pre-surgery.

## Author contributions

XS: Conceptualization, Data curation, Formal analysis, Methodology, Validation, Writing – original draft, Funding acquisition, Investigation, Resources, Software, Visualization. SS: Formal analysis, Validation, Writing – original draft, Writing – review & editing, Supervision. LW: Data curation, Formal analysis, Methodology, Software, Validation, Writing – original draft. JQ: Formal analysis, Validation, Writing – original draft, Data curation, Methodology, Software. RY: Data curation, Formal Analysis, Methodology, Software, Validation, Writing – original draft. JL: Conceptualization, Project administration, Supervision, Validation, Writing – original draft. JZ: Conceptualization, Supervision, Validation, Writing – original draft. EH: Conceptualization, Supervision, Validation, Data curation, Formal analysis, Methodology, Writing – original draft, Writing – review & editing. JPZ: Formal analysis, Methodology, Software, Validation, Conceptualization, Funding acquisition, Investigation, Project administration, Resources, Supervision, Visualization, Writing – original draft, Writing – review & editing
